# Technological tools for the measurement of sensory characteristics in food: A review

**DOI:** 10.12688/f1000research.131914.2

**Published:** 2024-02-01

**Authors:** José D Martinez-Velasco, Annamaria Filomena-Ambrosio, Claudia L Garzón-Castro

**Affiliations:** 1 Engineering Faculty - Research Group CAPSAB, Universidad de La Sabana, Campus del Puente del Común, Km 7 Autopista Norte de Bogotá, Chia, Cundinamarca, 250001, Colombia; 2 International School of Economics and Administrative Science - Research Group Alimentación, Gestión de Procesos y Servicio de la Universidad de La Sabana Research Group, Universidad de La Sabana, Campus del Puente del Común, Km 7 Autopista Norte de Bogotá, Chía, Cundinamarca, 250001, Colombia

**Keywords:** Sensorial characteristic, technological tools, electronic nose, electronic tongue, artificial vision, texture analyzer, acoustic analysis, food sector

## Abstract

The use of technological tools, in the food industry, has allowed a quick and reliable identification and measurement of the sensory characteristics of food matrices is of great importance, since they emulate the functioning of the five senses (smell, taste, sight, touch, and hearing). Therefore, industry and academia have been conducting research focused on developing and using these instruments which is evidenced in various studies that have been reported in the scientific literature. In this review, several of these technological tools are documented, such as the e-nose, e-tongue, colorimeter, artificial vision systems, and instruments that allow texture measurement (texture analyzer, electromyography, others). These allow us to carry out processes of analysis, review, and evaluation of food to determine essential characteristics such as quality, composition, maturity, authenticity, and origin. The determination of these characteristics allows the standardization of food matrices, achieving the improvement of existing foods and encouraging the development of new products that satisfy the sensory experiences of the consumer, driving growth in the food sector. However, the tools discussed have some limitations such as acquisition cost, calibration and maintenance cost, and in some cases, they are designed to work with a specific food matrix.

**Abbreviations**
a.u.: Acoustic Energy
ANN: Artificial Neural Networks
AVS: Artificial Vision System
CP: Conductive Polymers
CVS: Computer Vision System
DFA: Discriminant Function Analysis
EMG: Electromyography
GC-MS: Gas Chromatography-Mass Spectrometry
GC-O: Gas Chromatography-Olfactometry
HS-SPME: Headspace Solid Phase Microextraction
ICA: Imperialist Competitive Algorithm
LDA: Linear Discriminant Analysis
LEDs: Light Emitting Diodes
MOS: Metal Oxide Semiconductors
MSE: Mean Square Error
PCA: Principal Component Analysis
PLS-DA: Partial least square-discriminant analysis
PVC: Polyvinyl chloride
QCM: Quartz Crystal Microbalance
RGB: Red Green Blue
RSM: Response Surface Methodology
SAW: Surface Acoustic Waves
SVM: Support Vector Machines
VOCs: Volatile Organic Compounds

## 1. Introduction

The world of the food industry search to ensure satisfactory multisensory experiences for consumers through the consolidation of quality standards for food products ( Blissett & Fogel, 2013; Tuorila & Hartmann, 2020). The first approach to each food matrix allows the consumer to identify attributes related to size, shape, color, and brightness. A second approach allows more direct interactions related to the perception of smell, aroma, taste, temperature, and texture of the product ( Fine & Riera, 2019; Isogai & Wise, 2016; Moding *et al.*, 2020; Nederkoorn *et al.*, 2018). Recognizing these sensory characteristics determines the acceptance or rejection of the food ( Costell *et al.*, 2009; Torres Gonzalez *et al.*, 2015; Wadhera & Capaldi-Phillips, 2014). One of the disciplines that study the sensory characteristics of food is sensory analysis. This term became a field of study in the 17th century when Jean Anthelme Brillat-Savarin, in 1825, wrote his first book entitled Philosophy of Taste, in which he established the basis for the analysis of food and how it is perceived ( Chong, 2012). The constant evolution of the concept and applicability of sensory analysis has consolidated its study using trained panelists or instrumental methods. Although the analyses carried out by these panelists constitute an essential source of information for the acceptance or rejection of a food product, this can be subjective due to biological, social, and other external factors surrounding the subject ( Buratti *et al.*, 2018; Loutfi *et al.*, 2015; Tan & Xu, 2020).

One of the main limitations when implementing sensory tests is the number of required panelists, ranging from 7 to 100 depending on the test type ( Lawless & Heymann, 2010; O’Mahony, 2017). This implies an investment of human and economic resources, raw materials, and/or time. This limitation has motivated researchers to generate technologies to identify and quantify some sensory characteristics of foods with greater precision ( Akimoto *et al.*, 2017; Kusumi *et al.*, 2020; Pascual *et al.*, 2018).

Such developments search to mimic the functioning of the five senses, such is the case of electronic noses (e-noses) and tongues (e-tongues), which upon contact with food, generate an electronic response from a chemical interaction, which is interpreted by a digital information processing system ( Banerjee *et al.*, 2019; Bonah *et al.*, 2020). Similarly, image analysis through devices such as cameras seek to simulate the sense of eyesight ( Ansari *et al.*, 2021; Barbon *et al.*, 2017; Kakani *et al.*, 2020; Khojastehnazhand & Ramezani, 2020); concerning touch and hearing, some reports show various technological tools that measure force and sound, seeking to imitate the behavior of these senses ( Akimoto *et al.*, 2019; Kato *et al.*, 2017; Kusumi *et al.*, 2020).

Each of the technological tools mentioned above contributes a description of the primary sensory characteristics of the food matrix to be evaluated. Few works show the use of more than one technological tool, despite the fact that the combination of these tools allows for better management of different types of resources (scientific personnel, economic resources, time, raw materials). However, Huang *et al.* (2023), Chen *et al.* (2023), Gao *et al.* (2022) and Martínez-Velasco *et al.* (2022) made use of more than one technological tool. This article consolidates information on some technological tools reported in the literature for sensory analysis in various food matrices.

## 2. Electronic nose (e-nose)

Odor is one of the most representative attributes of food. This can be expressed as one of the qualities of Volatile Organic Compounds (VOCs), so unique and distinctive that they are considered fingerprints ( Bonah *et al.*, 2020; Tan & Xu, 2020).

Generally, the sensory analysis method to identify such components is performed by panelists who rate and classify on different scales the odor perceived in the sample ( Barbieri *et al.*, 2021; Giungato *et al.*, 2018; Niu *et al.*, 2019; Świąder & Marczewska, 2021). On the other hand, different methods have been developed for the identification of VOCs, such as: 1) Gas Chromatography-Olfactometry (GC-O): this methodology is to assess the odor impact of volatile compounds present in a sample extract and assign a degree of significance to each individual compound. GC-olfactometry, or GC-O, encompasses a range of techniques that rely on human assessors to detect and assess the volatile compounds released during a gas chromatography separation ( Delahunty *et al.*, 2006). 2) Gas Chromatography-Mass Spectrometry (GC-MS): is an analytical technique that integrates the capabilities of both gas chromatography and mass spectrometry to detect and identify various substances present in a given test sample. This technique is utilized within flavor research to identify the aroma-contributing compounds in various food products. These methodologies encompass approaches such as dilution analysis, detection frequency techniques, posterior intensity assessments, and time-intensity evaluations ( Van Ruth, 2001). 3) Headspace Solid Phase Microextraction (HS-SPME), is a modern and highly sensitive sample preparation technique that does not require solvents. HS-SPME has emerged as a potent sample preparation method that efficiently enables the isolation and concentration of analytes from intricate matrices. It utilizes a coated fiber to concentrate volatile and semi-volatile compounds from a sample, operating on the principles of adsorption/absorption and subsequent desorption ( Lancioni *et al.*, 2022).

These methods (GC-O, GC-MS, HS-SPME) are characterized by high accuracy and reliability, as some of the most used methods ( Attchelouwa *et al.*, 2020; Chen *et al.*, 2021). However, these methods usually require sample conditioning, which involves investing many different types of resources ( Shi *et al.*, 2018). Considering the above, devices such as the e-nose have been developed, consisting of an array of electrochemical sensors articulated with a pattern recognition system that identifies, groups, and discriminates the VOCs ( Gliszczyńska-Świgło & Chmielewski, 2017; Loutfi *et al.*, 2015). This has become an alternative to generating fast and reliable results in the food industry ( Barbosa-Pereira *et al.*, 2019; Conti *et al.*, 2021; Wasilewski *et al.*, 2019).

### 2.1 The internal structure of the e-nose

**Figure 1.**
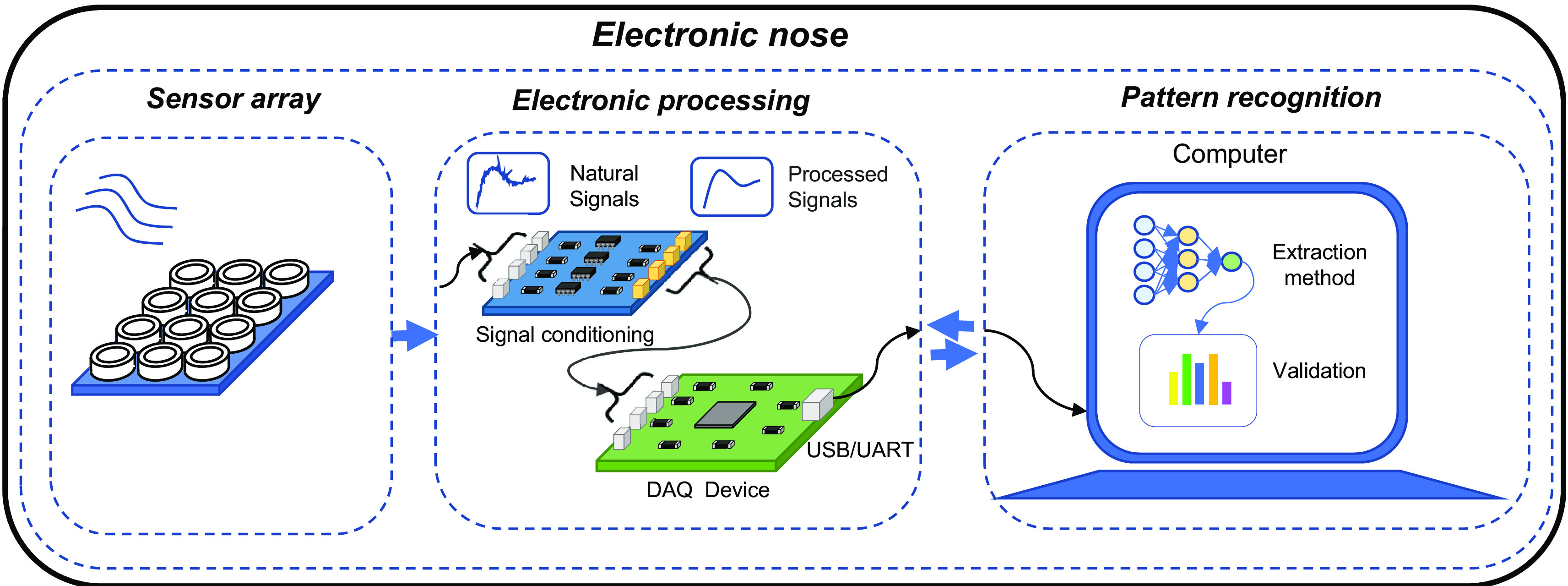
Fundamental stages of operation of an electronic nose.

The e-nose is a device that seeks, like humans, to perceive, identify and classify odors. The process carried out by an e-nose compared to the human nose can be described as follows: the odor molecules are exposed to the e-nose (which corresponds to the human nose), the chemical patterns present in the sample of the aroma are detected by sensors (which are equivalent to the olfactory receptor neurons), which transform this chemical input into an electrical signal, producing, for each aroma, a unique response pattern, designated as an olfactory fingerprint (function performed by the olfactory bulb). Finally, pattern recognition techniques are applied to this response to discriminate, classify and/or predict the type of aroma being analyzed (action developed in the brain thanks to neurons) ( Moreno *et al.*, 2009). Thus, the e-nose is characterized by the articulation of three fundamental systems: sensing, electrical conditioning, and pattern recognition; see Figure 1.

The sensing system is composed of a matrix of sensors that can be of different types such as: conductivity, polymers, Conductive Polymers (CP), Metal Oxide Semiconductors (MOS), Surface Acoustic Waves (SAW), and Quartz Crystal Microbalance (QCM), which allow the detection of VOCs through absorption, adsorption, or chemical reaction methods. Depending on the characteristics of the food matrix to be evaluated, the sensors that make up the e-nose must be carefully considered, as they will react more efficiently to certain particles ( Tan & Xu, 2020; Wilson & Baietto, 2009). This detection produces an electronic signal, from which it is possible to characterize the VOCs.

The electrical conditioning system is responsible for matching the signal emitted by each of the sensors. Signal matching consists of amplification and filtering to identify the analyzed food matrix sample ( Shi *et al.*, 2018).

Finally, the pattern recognition system receives the already conditioned electrical signal and is in charge of processing it. For this procedure, extraction methods are used, which aim to obtain reliable and robust information from the electrical signal, guaranteeing greater measurement efficiency. Some extraction methods are: Principal Component Analysis (PCA), Support Vector Machines (SVM), Artificial Neural Networks (ANN), Linear Discrimination Analysis (LDA), Discriminant Function Analysis (DFA), decision trees, and other machine learning classifiers ( Tan & Xu, 2020; Yan *et al.*, 2015).

### 2.2 E-nose applications

E-nose is used in several food matrices to identify their authenticity due to the growing number of counterfeit products that represent a significant risk to the health of consumers ( Gliszczyńska-Świgło & Chmielewski, 2017). Additionally, this device also allows users to identify and group according to their specifications some food matrices such as: alcoholic beverages, dairy products, and juices ( Sanaeifar *et al.*, 2017); the ripeness of fruits and vegetables; quality of meats; shelf life of grains, among others ( Du *et al.*, 2019; Tan & Xu, 2020; Wang *et al.*, 2019).

For example, the e-nose of the Alpha MOS FOX family has been used to identify possible adulteration of olive oil with hazelnut and sunflower oils ( Mildner-Szkudlarz & Jeleń, 2008). Also, in the analysis of flaxseed oil detecting adulteration with other similar components ( Wei *et al.*, 2015).

In research conducted by Nurjuliana (2011), the volatile compounds in pork, beef, lamb, and chicken sausages were analyzed. The samples taken from each of the sausages were analyzed by mass spectrometry, gas chromatography, and zNose™ electronic nose, which allowed the identification of the type of meat from which the sausages were made. Although the results of the tests carried out by all the instruments were highly efficient, the speed and low cost of using the zNose™ e-nose were highlighted.

Additionally, in the research by Ghasemi-Varnamkhasti *et al.* (2019), an e-nose was custom designed using five types of MOS sensors to classify two pieces of cheese: Roquefort and Camembert. This classification was carried out by taking into account the milk (sheep, goat, or cow) with which it was made, the degree of pasteurization, and the maturity of these cheeses.

Other reports show the use of e-noses to analyze fish. Güney and Atasoy (2015), used a low-cost e-nose developed at Karadeniz University, composed of 8 metal oxide gas sensors, to classify three fish species (Horse mackerel ( *Trachurus murphyi*), Anchovy ( *Engraulidae*) and Whiting ( *Merlangius merlangus*). In addition, Zhang *et al.* (2012a), analyzed VOCs during the storage and freezing process of sawfish ( *Scomberomorus niphonius*), finding a linear relationship between a volatile nitrogen base with triethylamine. A separate investigation reports the use of the commercial e-nose Alpha MOS FOX 3000, composed of 18 MOS-type sensors, to establish the sensory profile of the active aromatic compounds of cumin ( *Cuminum cyminum* L.) ( Ravi *et al.*, 2013).

Table 1 shows some relevant studies using e-nose in the food, specifying: product, purpose of the analysis, e-nose model, type of sensor, extraction method, and main result obtained.

**Table 1.** Results of relevant studies using electronic noses in the food industry.

Food	Purpose of the analysis	Electronic nose model and combinations	Sensor type	Extraction method used	Results	Reference
Meat Floss	Identify the origin of meat floss (beef, pork or chicken) by building an e-nose and implementing a supervised machine learning method	Custom Design	Eight (8) MOS sensors	Four (4) different supervised learning methods: LDA, QDA, k-nearest neighbors (k-NN), and random forest (RF).	Highest accuracy values of >99% for both validation and testing data in discriminating beef, chicken, and pork flosses	( Ardita Putri *et al.*, 2023)
*Terfezia arenaria*	To show the nutritional and chemical composition, as well as the volatile profile of *T. arenaria*	Eletronic nose Cyranose-320 (Sensigent, Pasadena, CA, USA)	32 sensors	N/A	The Cyranose-320 correctly classified 73% of the T. areanaria samples front other edible mushrooms and truffles (A. bisporus, L. edodes, P. ostreatus and T. melanosporum) incubated at room temperature, and 81% of the T. areanaria samples incubated at 40 °C	( Ferreira *et al.*, 2023)
Jams production	Develop a system that is able to detect the mold contamination on fruit and vegetable jams and marmalades	Custom Design	Six (6) MOS sensors	PCA	An anomaly detector capable of recognizing the appearance of possible contamination (various samples of fruit and vegetable preparations), thus acting as an early warning system in the food chain	( Greco *et al.*, 2023)
Bee pollen	To evalued the sensory consistency of moist pollen, pollen dried in the sun, and pollen dried in a controlled environment while subjecting them to accelerated storage at temperatures of 30, 40, and 50°C	EN3 (AIRSENSE Analytics GmbH, Schwerin, Germany)	10 semiconductor sensors array	PCA	Bee pollen samples with a high water activity showed VOC profile major changes during storage as well as their colour change. Bee pollen samples with a low water activity presented a change in their smell associated with fat rancidity, which is directly related to the texture	( Correa *et al.*, 2022)
Cheese	Analysis of cheese ripening with raw and pasteurized milk	Custom Design	Six (6) piezoelectric quartz crystals	PCA and PLS-DA	Discrimination of cheeses of each milk type	( Valente *et al.*, 2018)
	Comparison of aroma intensity to sensory measurement	POLFA	MOS	N/A	Demonstrated a linear correlation between the two factors (Pearson’s R = 0.983)	( Fujioka, 2021)
	Origin and authenticity of Oscypek cheese with Protected Designation of Origin (PDO)	SPME-MS	MS	PCA, LDA, SIMCA, SVM	Classification between 90% and 97% according to the extraction method	( Majcher *et al.*, 2015)
Sesame Oil	Identify adulterated sesame oil by means of aroma measurements.	Custom Design	Nine (9) MOS sensors	SVM, ANN	The sensitivity and specificity obtained for SVM were 98.7% and 97.7%, respectively, while these values for the ANN method were 94.9% and 95.3%, respectively	( Aghili *et al.*, 2023)
Argan oil	Identification of adulteration with sunflower oil	MOS electronic gas nose	Five (5) MOS sensors	PCA, DFA, SVM	85% identification of original oil and 87% identification of adulterated oil	( Bougrini *et al.*, 2014)
Flaxseed oil	Oils processed differently for counterfeit detection	Alpha MOS FOX 3000	18 MOS sensors	PCA	87% success rate in counterfeit detection	( Wei *et al.*, 2015)
Pork	Identification of adulteration of minced pork with spoiled pork	PEN 2	10 MOS sensors	CDA, BDA, PLS, MLR, and BPNN	The identification success rate of 97%	( Tian *et al.*, 2013)
Ham	Differentiation of PDO marked hams	PEN 2	10 MOS sensors	PCA	Differentiation between ham types between 80% and 87%	( Laureati *et al.*, 2014)
Honey	Sugar beet and sugar cane adulteration identification	Cyranose320	32 sensors of different types of polymeric matrix, mixed with carbon black	ANN	Identification of samples with a success rate of 89.5%	( Subari *et al.*, 2014)
	Confirmation of botanical origin	Alpha MOS Fox 4000	18 MOS sensors	PCA, DFA, LS-SVM, PLS	The success rate is between 81% and 90%, depending on the extraction method	( Huang *et al.*, 2015)
	Confirmation of botanical origin and identification of adulteration with rice and corn syrups	Flash GC	--	PCA, SVM, PLS	Difference between samples with a 71% success rate and a 65% success rate in identification	( Gan *et al.*, 2016)
Cherry tomato juice	Identification of adulteration with ripened tomato juice	PEN 2	10 MOS sensors	PCA, CA	Identification with a 76% success rate	( Hong *et al.*, 2014)
Spirits	Confirmation of botanical origin (rye, triticale, wheat, distilled agricultural corn)	Flash GC	--	PCA, DFA, SIMCA, SQC	The success rate is between 71.9% and 82.9% depending on the extraction method	( Wiśniewska *et al.*, 2016)
Liquor	Identification of authenticity of traditional Polish beer Nalewka	Flash GC	--	PCA, DFA, SIMCA, SQC	Identification with a success rate between 22% and 89.5% depending on the sample and extraction method	( Śliwińska *et al.*, 2016)
Peach	Impairment detection	Fox 4000	18 MOS sensors	PLSR, LS-SVM, MFRG	A prognostic model of fruit decay was obtained with a response rate of 82.26%	( Huang *et al.*, 2017)
Bell pepper	Freshness evaluation	iNose (Ruifen Trading Co)	14 MOS sensors	HCA, PCA, PLS	Differentiation in the days after harvest was obtained. Obtaining a statistical model of (R ^2^ = 0.9783, RMSE = 0.3317)	( H. Z. Chen *et al.*, 2018)
Cocoa	Fermentation degree detection	Custom Design	Six (6) MOS sensors	ANN	9.4% misclassification rate	( Tan *et al.*, 2019)
Rice	Detection of infection in rice	PEN2	10 MOS sensors	PCA and PLSR	Prediction result of Rp ^2^ = 0.864 and RMSEP = 0.235	( Gu *et al.*, 2020)
Dragon fruit, Snow pear, Kiwi fruit, and Fuji apple	Determination of freshness and degradation	Custom Design	Eight (8) MOS sensors	PCA	Discrimination of four levels of fruit condition between 91.12% and 93.69% in the PCA	( Ding *et al.*, 2018)

## 3. Electronic tongue (e-tongue)

The human tongue can identify five basic tastes: sour, salty, sweet, bitter, and umami ( Beauchamp, 2019). Usually, the evaluation and classification of the basic flavors of a product are done through trained panelists and sometimes consumers ( Jiang *et al.*, 2018). However, these measurements can be subjective, which can be reduced by using technological tools such as the e-tongue, thus ensuring repeatability and reproducibility of the results ( Schlossareck & Ross, 2019). Ross (2021) showed that combining different electrodes makes it possible to identify different flavors, such as fatty, metallic, and others. Different investigations have shown that by using the e-tongue, it is possible to determine the quality, adulteration, classification, or origin of food ( de Morais *et al.*, 2019; Elamine *et al.*, 2019; Jiang *et al.*, 2018; Sobrino-Gregorio *et al.*, 2018). The previously mentioned characteristics have allowed the e-tongue to become a fast, economical and impartial detection alternative ( Titova & Nachev, 2018); this is because it allows the characterization of the flavor of the food matrix ( di Rosa *et al.*, 2017). Additionally, the e-tongue has a matrix of electrodes that, according to their combination and characteristics, produce potentiometric, voltametric, and impedimetric signals ( Jiang *et al.*, 2018).

### 3.1 The internal structure of the e-tongue

**Figure 2.**
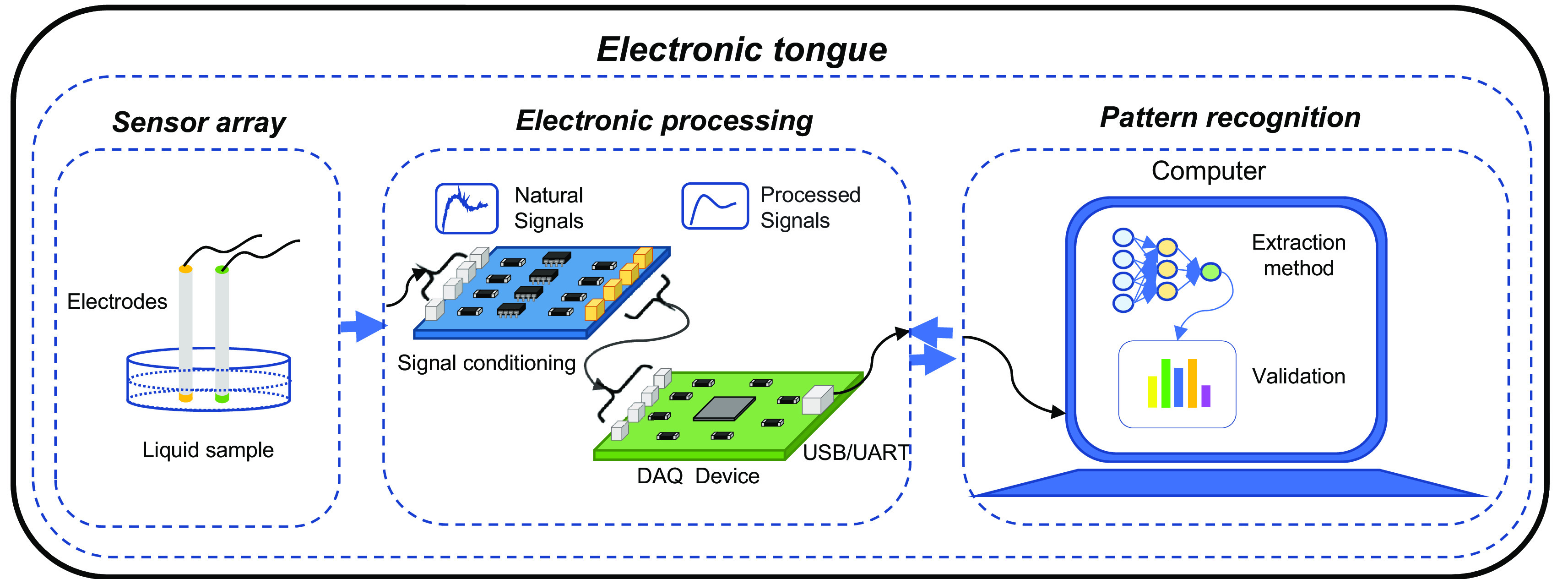
Fundamental stages of operation of an electronic tongue.

The process carried out by an e-tongue compared to the human tongue can be described as follows: the liquid or food comes into contact with the sensor system (which corresponds to the taste cells spread on the tongue), the chemical patterns present in the sample are detected by the sensors (which is equivalent to the stimulation of taste cells), which transform this chemical input into an electrical signal, producing to which a pattern analysis algorithm is applied to discriminate, classify and/or predict the type of flavor being analyzed (action developed by the neurons in the brain where the flavor is recognized) ( Tan & Xu, 2020; Arrieta *et al.*, 2010). Thus, e-tongue is characterized by articulating three fundamental systems: sensing, electrical conditioning, and pattern recognition ( di Rosa *et al.*, 2017) (see Figure 2).

E-tongue sensing system is composed of two or more electrodes, each electrode has a membrane that upon contact with the analyte generates a chemical interaction causing a reversible change in the electronic properties, which allows the characterization of the food matrix ( Tan & Xu, 2020).

Potentiometric-type electrodes measure the voltage differences between the working and the reference electrodes ( Wasilewski *et al.*, 2019). The voltage change in the measurement given by the working electrode will have a proportional relationship to the concentration of the analyte ( Jiang *et al.*, 2018; W. Wang & Liu, 2019). Some of the membranes used in potentiometric electrodes can be multi-channel lipid with a reference electrode made of a silver/silver carbon alloy (Ag/AgC), chalcogenide glass with a polyvinyl chloride (PVC) film, liquid or polymeric, which allow the detection of the voltage generated when in contact with the food matrix ( Tan & Xu, 2020).

Regarding voltametric electrodes, these are used in conjunction with a minimum electrode configuration in which one must have a working, a reference, and an auxiliary electrode ( Jiang *et al.*, 2018; Wasilewski *et al.*, 2019). Generally, these working electrodes are constituted by a bare or modified metal, which contemplates any of the following compounds: copper (Cu), nickel (Ni), palladium (Pd), silver (Ag), tin (Sn), titanium (Ti), zirconium (Zr), gold (Au), platinum (Pt) and radium (Ra) ( Jiang *et al.*, 2018). Its operation encourages the transfer of electrons through the food matrix, measuring the resulting polarization current, which has a direct relationship with the concentration of certain components present in the food ( Wei *et al.*, 2018).

Another group of electrodes is those of impedimetric type, characterized by being coated with different polymeric materials, which, upon receiving an alternating signal of variable frequency and constant amplitude, produce an alteration in the impedance value ( Garcia-Hernandez *et al.*, 2018). This impedance change allows for characterizing, detecting, and discriminating different components such as: sucrose (C _12_ H _22_ O _11_), sodium chloride (NaCl), potassium chloride (KCl), and hydrochloric acid (HCl) ( Podrazka *et al.*, 2017). According to the literature, the most used electrodes on the market are potentiometric and voltametric electrodes due to advanced development ( Wang & Liu, 2019).

Tan and Xu (2020) indicated that electrodes in the development phase incorporate biomaterials such as enzymes, whole cells, tissues, receptors, or antibodies, whose chemical interaction with the food generates a transfer of electrons, ions, or molecules. This transfer modifies the characteristics of the electronic signal, like those produced by potentiometric and voltametric electrodes. It is expected that these biosensors will be a technology that will contribute to improving results in the future.

The electrical conditioning and pattern recognition systems of the e-tongue present particularities closely like those of the e-nose. The only substantial difference between these two technological tools is presented in the sensing system in terms of the characteristics specific to the internal and structural design of the sensors ( Tan & Xu, 2020; Wasilewski *et al.*, 2019).

### 3.2 E-tongue applications

The use of the e-tongue in the food industry encompasses a wide range of applications, including discrimination by type and place of origin, verification of authenticity, adulteration or counterfeiting, and quantification of food matrix components ( Titova & Nachev, 2018; Wasilewski *et al.*, 2019).

A clear example of the use of such technology for classifying products by type and place of origin is evidenced in the research developed by Souayah (2017), where a potentiometric e-tongue was used to classify 60 samples of olive oil. Moreover, Elamine *et al.* (2019) discriminated 31 samples of honey from Portugal by botanical origin using an impedimetric e-tongue.

Cetó and Pérez (2020) used an inset voltametric e-tongue from Bas Inc. configured with three electrodes of gold (Au), platinum (Pt), and glassy carbon (C), to carry out the process of identification of authenticity and classification of 44 samples of six different varieties of vinegar. The measurement results of the equipment were subjected to the PCA and LDA extraction methods, which allowed the discriminating and categorizing of the total of the analyzed samples with 100% accuracy. This research allowed it to generate records of the electrochemical fingerprints of the vinegar.

Furthermore, a voltametric-type e-tongue was custom-developed to identify adulteration in roasted ground coffee ( de Morais *et al.*, 2019). This research analyzed 90 cups of coffee (60 unadulterated and 30 adulterated). LDA, SPA, and PLS-DA identification methods were applied to the measurements obtained; as a result, the adulterated beverages were identified and the purity percentage in each sample was quantified.

Another example is the investigation of the evolution process of taste compounds in the chicken stew at different cooking times, which focused on detecting nucleotides and free amino acids using a commercial e-tongue (TS-5000Z, Insent). As a result, the proportion of the components detected in each cooking stage and the identification of inosine monophosphate (IMP), glutamic acid (Glu), lysine (Lys), and sodium chloride (NaCl) as the main compounds highlighted the final flavor attributes of the chicken were evidenced ( Liu *et al.*, 2017). Table 2 shows some relevant studies in which e-tongues in different food matrices.

**Table 2.** Results of relevant studies using electronic tongues in the characterization and identification in the food matrices.

Food	Purpose of the analysis	Type of electrode used in the electronic tongue	Extraction method	Results	Reference
Hanwoo beef (crossbreed between Bos taurus and Bos zebu)	To used three different feed types and investigated their effects on Hanwoo quality by analyzing the color, texture, fatty acid content, and amino acid content of meat	Biomimetic membrane (TS-5000Z, Kanagawa, Japan)	PCA	The e-tongue analysis results were strongly correlated with the human sensory evaluation findings of umami taste. Hanwoo’s umami flavor	( Min *et al.*, 2023)
Baked food (brownie)	To evaluate the possibility to add fractions recovered from residues of orange, lime, and peach palm in a baked food	Biomimetic membrane (TS-5000Z, Inset)	One-way Analysis of Variance (ANOVA), Tukey test	This study showed the great potential of using fruit residues in the food industry to enhance their functional properties and design healthier products sustainably	( Durán-Aranguren *et al.*, 2023)
Soup	To investigate using split-gill mushroom (SGM) powder containing umami taste to increase saltiness in a clear soup for two different heating conditions	Conductivity (α-ASTREE, Alpha MOS Company)	PCA	The addition of SGM and volumetric microwave heating could be an alternative method to reduce the amount of salt in soup by increasing umami flavor intensity and salinity	( Hiranpradith *et al.*, 2023)
Cheese	To develop a new Japanese cheese having different characteristics than the other mold–ripened cheeses	Lipid membrane (TS-5000Z, Intelligent Sensor Technology Inc.)	PCA	The analysis showed that koji-ripened cheeses have unique flavor characteristics compared to commercial Camembert cheese	( Hayashida *et al.*, 2023)
Milk	Brand Classification	Voltametric	PCA and PLS	80.5% success rate	( Yu *et al.*, 2015)
	Quantitative analysis of urea in adulterated milk	Voltametric	PCA and PLS	Identification and separation of different components	( Li *et al.*, 2015)
Ham	Measurement of curing processes with different amounts of salt	Potentiometric	RNA	Differentiation with a 100% success rate	( Gil-Sánchez *et al.*, 2015)
	Comparison of umami flavor peptides in water-soluble extractions	Voltametric	PCA	Comparison with 65% success rate	( Dang *et al.*, 2015)
Meat	Quality modeling and classification by breed	Potentiometric	PCA and LDA	100% identification and 97.5% prediction for each breed	( Surányi *et al.*, 2021)
	Ammonia and putrefaction detection	Voltametric	PCA and PLS-DA	Classification of samples with ammonia at 100%	( Apetrei & Apetrei, 2016)
Pork	Determination of the role of salt in the flavor of the meat	Lipid Membrane	PCA	Identification of the highest flavor indexes in dry-cured meat with a salt content of 3% and 5%	( Tian *et al.*, 2020)
Vegetable oil	Determination of three quality parameters	Potentiometric	PCA and PLS	Quantification of the three parameters with a relative error of 20%	( Semenov *et al.*, 2019)
Vegetable milk	Emulation of sensory analysis for product discrimination	Voltametric	PCA and PLS	Product differentiation with a variance of 77%	( Pascual *et al.*, 2018)
Red Wine	Evaluation of phenolic contents for 14 varieties of liquor	Voltametric	PCA and PLS	Validation with a variance of 85.8%	( Garcia-Hernandez *et al.*, 2020)
Honey	To study the effect of different temperature and time intervals on physicochemical parameters of honey. Using fusion between near infrared spectroscopy (NIRS) and electronic tongue (ET)	Potentiometric (Alpha MOS, Toulouse, France)	PCA, LDA	The model of the fused dataset provided >98% average correct classification of the models and 100% correct classification of the control honeys	( Bodor *et al.*, 2023)
	Validation of adulteration	Voltametric	PLS-LDA, LSD and MLR	Classification of samples between original and adulterated with an accuracy of 97.5%	( Oroian *et al.*, 2018)
Tea	Classification of different species	Voltametric	LDA, SPA, GA and SW	100% success rate classification with LDA/SPA method	( Rodrigues *et al.*, 2018)
	Measurement of phenolic compounds during the storage process for quality assurance	Potentiometric	PLS	Classification of the different types of tea with a coefficient of determination of Rp ^2^ between 0.926 and 0.956	( Ruengdech *et al.*, 2019)
Blueberry juice	Characterization of four types of cranberry juice for flavor profiling	Potentiometric	ANOVA and PLS	Characterization of flavor profile components given a cross-correlation with a variance of 83.14%	( Yu *et al.*, 2018)
Honey	Discrimination of botanical origin	Impedimetric	PCA	Discrimination of each characteristic of honey types	( Elamine *et al.*, 2019)
Red Meat and Poultry	Determination of optimal dilution level of meat extract	Potentiometric	LDA	Discrimination with an accuracy between 68.77% and 78.13%, depending on the dilution percentage	( Zaukuu *et al.*, 2021)

## 4. Colorimeter and Artificial Vision System

### 4.1 Colorimeter

A colorimeter is a sensor device used to measure color in different surfaces and liquids ( Anzalone *et al.*, 2013). According to Millikan (1993), a colorimeter can detect different shades of colors by analyzing the reflection of light in different objects. The principle by which the colorimeter works is the emission of light over the material that must be analyzed and the corresponding reading of the reflection of color. The device then emits a code, representing the exact shade measured. Anzalone (2013), mentions that colorimeters are widely used in food industry, medical procedures, and demographic measurement of protozoa.

An essential aspect of working with digital images revolves around information processing. This is because cameras capture RGB (Red, Green, and Blue) values that must be converted into the CIELAB color space.

Table 3 shows some relevant studies using colorimeter in the food, specifying: product, device, and results.

**Table 3.** Results of relevant studies using colorimeter in the characterization and identification in the food matrices.

Food	Device	Results	Reference
Bread	CR-400 colorimeter, Konica Minolta	Enriched bread with CTS at a cellular level showed significant decreases in the values of a* and b* during storage. The addition of CTS at a cellular level helped prevent changes in L* and b*, achieving better control of bread aging and maintaining product quality for a longer period.	( Wang, L. *et al.*, 2023)
Pork Meat	CR-400 colorimeter, Konica Minolta	Meats cooked using vacuum and sous vide methods were observed to have a lighter appearance, which is associated with elevated L* color values. Vacuum cooking also resulted in a greater hue angle and reduced chroma.	( Ángel-Rendón, S.V. *et al.*, 2020)
Cricket flour and traditional beverage *(chucula)*	CR-400 colorimeter, Konica Minolta	Different changes in color coordinates	( Sotelo-Díaz, L.I. *et al.*, 2022)
Cocoa Seed	14.2-megapixel Sony α380 digital camera (Sony, Japan)	To establish a correlation between epigallocatechin content and four color parameters. In this way, color image analysis could be an appropriate alternative to predict the concentration of quality-related compounds in cocoa matrices.	( Becerra, L.D. *et al.*, 2023)
Cabernet Sauvignon wines	UV spectrophotometer (Shimadzu, Tokyo, Japan)	As the harvest ripeness elevated, wine’s flavonoid profiles were altered and gained a higher red color intensity.	( Lu, H.C. *et al.,* 2023)
Milk and Milk Products	CR-400 colorimeter, Konica Minolta and a computer vision system (CVS).	Was difference between colour measured by CVS and the colorimeter; colorimeter readings resulted in a darker and yellower colour based on average L∗a∗b∗ values, while CVS readings resulted in lighter and less yellow appearance.	( Milovanovic, B. *et al.,* 2021)

### 4.2 Artificial Vision System

Computer Vision System (CVS) also known as Artificial Vision System (AVS), is an image analysis tool used to obtain information about objects through them ( Bhargava & Bansal, 2018; Wu & Sun, 2013). This is due to its ability to characterize: shape, size, color, and other particularities of the object, which can be static or moving ( Zhu *et al.*, 2021). Therefore, the CVS can be used in both continuous and static production lines, achieving a real-time analysis, as it allows fast, accurate, and non-invasive captures, with reliable and reproducible results ( Barbon *et al.*, 2017; Patrício & Rieder, 2018). Due to its flexibility and technological development, a CVS can store information about an object to perform further analysis using new images ( Taheri-Garavand *et al.*, 2019; Wu & Sun, 2013). Thus, the CVS becomes an alternative to avoid the possible errors of quality inspection of the objects which the human eye can incur ( Patrício & Rieder, 2018).

#### 4.2.1 CVS internal structure

**Figure 3.**
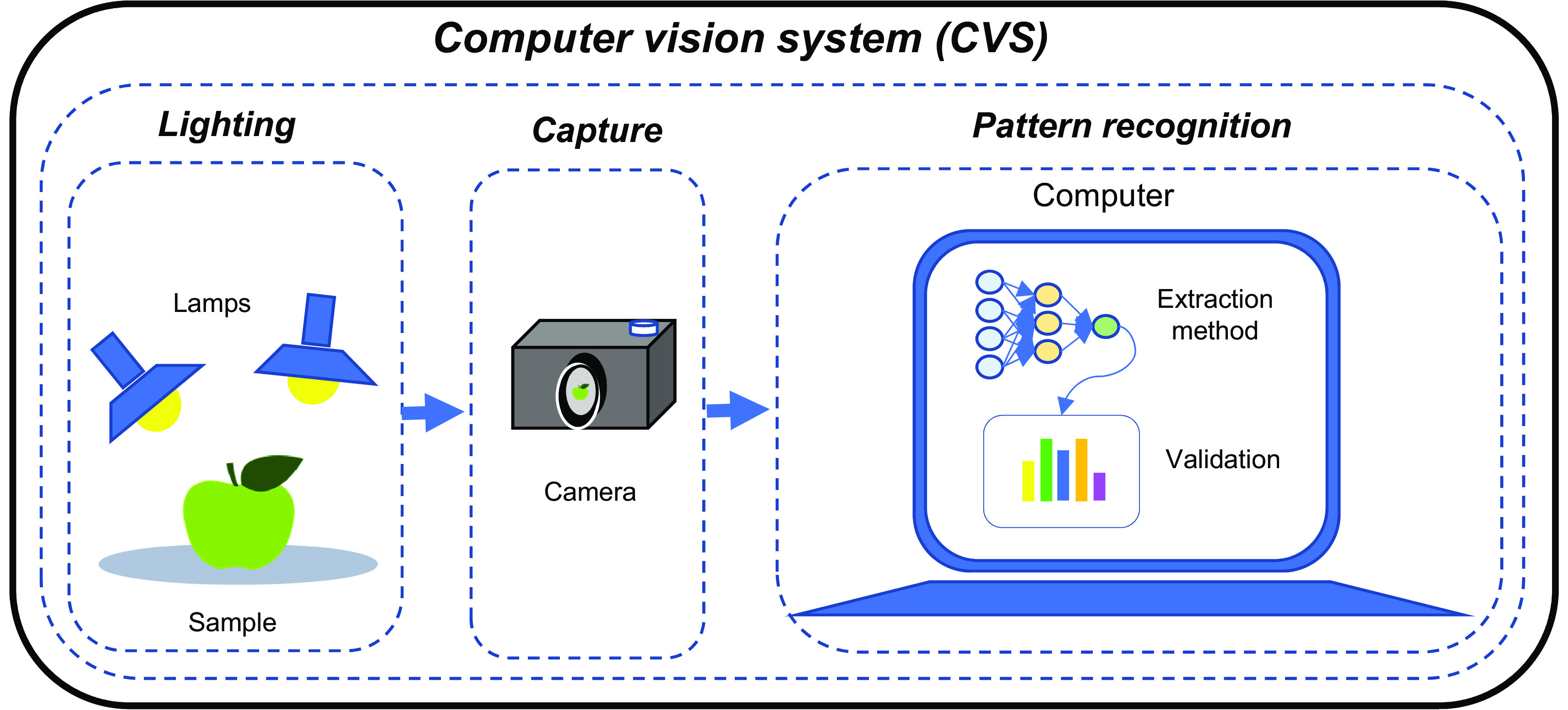
Fundamental stages of operation of a machine vision system.

A CVS is composed of three fundamental stages: illumination, image detection, and pattern recognition ( Kakani *et al.*, 2020), see Figure 3. The first stage plays an important role in image acquisition, since light has a direct impact on the clarity and color of the images and its improper use can generate shadows and unwanted reflections, cataloged as noise in the images ( Vithu & Moses, 2016). Therefore, depending on the application of the system, an appropriate selection of the light-generating elements must be made, considering characteristics such as wavelength, intensity, and direction. These light-generating elements can be light bulbs (incandescent, fluorescent, halogen), lasers, light emitting diodes (LEDs), X-ray tubes, and infrared lamps ( Naik & Patel, 2017; Sun *et al.*, 2019; Zhu *et al.*, 2021). These ensure clarity, repeatability, and reliability of the image ( Barbon *et al.*, 2017). This process is like that carried out by the human visual system, where light stimuli reach the cornea (the curved front layer of the eye that assists in focusing), which then focuses the light onto the pupil to enter the eye. However, the iris controls the amount of light that enters the pupil ( Frisby & Stone, 2010).

Two of the most used technologies in the second stage are cameras or scanners, which are responsible for taking an image of the object to be analyzed. Cameras capture a two-dimensional image instantaneously, while scanners take a line of pixels in an instant of time, so it requires a mechanism that performs a displacement of the scanner or the object to capture a succession of data and thus obtain the two-dimensional image ( Patrício & Rieder, 2018). Internally, these devices have specialized sensors that can capture color, monochromatic, thermal, or ultraviolet images depending on their characteristics ( Patrício & Rieder, 2018; Sun, 2016; Vithu & Moses, 2016; Zhang *et al.*, 2014). Other technologies used in this stage are: Hyperspectral, Magnetic Resonance, and X-Ray ( Sun, 2016; Zhang *et al.*, 2014). Drawing an analogy with the behavior of the sense of sight, this stage corresponds to when the lens collects the incoming light beam in the eye. The lens allows for focusing on objects and, along with the cornea, correctly focuses the light on the retina. This beam of light travels through the vitreous cavity (a hollow space filled with a transparent gel-like fluid that serves as the medium through which light travels from the lens to the retina). In the retina (which functions as a projection screen), thanks to the presence of photoreceptors (rods, responsible for peripheral and nighttime vision, and cones, sensitive to the color of light), the light information is converted into a nerve impulse that is sent to the cerebral cortex through the optic nerve ( Frisby & Stone, 2010).

Finally, the third stage aims to extract quantitative and qualitative information from the image using an analysis algorithm usually run on a processor ( Zhu *et al.*, 2021). Depending on the application and the complexity of the system, image processing is divided into three different levels: low, medium, and high. At the first level, operations such as cleaning of noise caused by shadows or external elements, quality enhancement, or correction of image illumination errors are performed ( Patrício & Rieder, 2018). Then, at the medium level, segmentation, description, classification of shapes, and image dimensions are performed ( Sun *et al.*, 2019; Taheri-Garavand *et al.*, 2019). Finally, at the third level, more complex operations are performed, including classification, comparison, and discrimination of the characteristics of the object in the image. These operations can be applied to the area or regions of interest using analysis methods such as statistical tools or computational models such as neural networks, which are some of the most used extraction methods ( Kakani *et al.*, 2020; Patrício & Rieder, 2018). When relating this phase to the sense of sight, the nerve signal that reaches the cerebral cortex is interpreted through a psychochemical process and transformed into an image ( Frisby & Stone, 2010).

Given the versatility and advantages presented by a CVS, the food industry has been implementing these systems to identify properties such as: morphology, color, texture, freshness, and quality ( Bhargava & Bansal, 2018; Patrício & Rieder, 2018; Taheri-Garavand *et al.*, 2019; Vithu & Moses, 2016). In general, the information collected is fed into databases to train learning algorithms and establish patterns to build a knowledge base, with which a system for autonomous decision-making can be implemented to provide an agile and flexible solution ( Zareiforoush *et al.*, 2015).

#### 4.2.2 CVS applications

The applications that recurrently use CVS are focused on the classification and prediction of the characteristics of a food matrix, whether it is an individual analysis, a production batch, or harvesting ( Arsalane *et al.*, 2020; Kakani *et al.*, 2020; Velesaca *et al.*, 2021). Research such as the one carried out by Arselane *et al.* (2020) in which they were able to successfully evaluate and determine the freshness of beef based on color and texture obtained by a portable custom-designed CVS. The system comprises fluorescent lighting, a GigEPRO camera, and an EVM6678 processing system in which PCA, SVN, PNN, and LDA algorithms were evaluated using Matlab ^®^. In a similar investigation carried out by Barbin (2016) to find the relationship between color and quality of chicken meat, a CVS was used with a Doc L-Pix camera.

Researchers such as Ghyar and Birajdar (2017), implemented a CVS, with which the state of pests in the rice plants was identified, to determine and discriminate anomalies or disease traits using leaf texture and color as reference parameters. The system developed consists of a Sony F470 camera, LED illumination, and computer analysis where ANN and SVM algorithms were run. Similarly, Koklu and Ozkan (2020) carried out the classification of seven different bean varieties to ensure the uniformity and quality of the seeds, identifying the characteristics of each bean species such as: area, perimeter, length of major and minor axes, aspect ratio, roundness, equivalent diameter, among others. The CVS was equipped with a Prosilica GT2000C camera, LED lighting, and a processor where an ANN algorithm was implemented in Matlab ^®^.

The research performed by Shrestha *et al.* (2016) reported a morphological analysis of wheat kernels to segment and classify them into three groups: healthy, damaged, and very damaged, as a consequence of premature germination. The result obtained was the segmentation and classification of the three groups of grains with an accuracy of 95% and 72.8%, respectively. The custom-designed system has two RL04C-OC cameras (Ximea GmbH, Germany), LED lighting system, and ANN implemented in Matlab ^®^.

Other applications of CVS systems are in fruits and vegetables, such as the one carried out by Santos Pereira (2018), where he classified the ripeness level of harvested papayas through the identification of color, length, diameter, and weight with an accuracy of 94.3% compared to manual classification. The CVS developed in-house, incorporates a Sony camera (Japan) located in an environment illuminated with white LED light. The pictures of each fruit were analyzed in Matlab ^®^ using a decision tree algorithm. Table 4 shows some relevant investigation where CVS has been used.

**Table 4.** Results of relevant studies with CVS in the characterization and identification of food matrices.

Food	Purpose of the analysis	CVS device	Attribute measured	Extraction method used	Results	Reference
Carrot slices	To implement a CV system in a prototype drier for real-time monitoring of product changes	Digital camera (mod. DFK 33UX264)	Size and color changes of carrot slices.	ANOVA	The CV system successfully tracked the shrinkage and colour changes of carrot slices during drying irrespective of pretreatments.	( Nallan Chakravartula *et al.*, 2023)
Raw pork loin	To develop a CV system to determine the color of a product	Digital camera (Sony Alpha DSLR-A200)	Color	Triangle tests and *d’*-value	This study show a systematic method to test consumers’ ability to differentiate between colors, variable that plays an important role in influencing consumer.	( Altmann *et al.*, 2022)
Potato ( *Solanum tuberosum*)	To determine the kinetics of color change in five varieties of potatoes, in the frying process	Digital camera (Canon SX 210)	Color	t-test	Describe the kinetics of browning (using RGB images) to stop the frying process at the right moment, also avoiding additional costs due to energy use, such as a final product with poor sensory quality.	( Salhuana *et al.*, 2022)
Milk	The CVS was compared with a colorimeter to identify similarities in the color measurement of twenty-seven different milks and milk products	Digital camera	Color	t-test and ANOVA	The comparison tests between the real color and the CVS indicated a similarity frequency of 100% in all cases	( Milovanovic *et al.*, 2021)
Apple	Detection of defective apples on a four-line fruit sorting machine Detection of defective apples on a four-line fruit sorting machine	RGB Camera	Color, size, and form	CNN	The model used get a performance of accuracy of 96.5%, recall of 100% and specificity of 92.9%, and accuracy of 92% for the testing set	( Fan *et al.*, 2020)
Tomatoes	Use of an ANN with a binary classification for the detection of external defects	CCD	Color and size	ANN	With the model used, they had an average precision of 97% on the test set, his optimal classified was 86.6% while maintaining a precision of 91.7%	( da Costa *et al.*, 2020)
Cherry tomato	Volume and mass estimation	Microsoft Kinect Camera	Size	SVM, Bayesian-ANN	The relation between tomato mass and volume was established as M1.312V^0.995 the mass was estimated at an R2 of 0.9824, with accuracy between 0.9226 and 0.9706	( Nyalala *et al.*, 2019)
Olive oils	Determine the moisture and insoluble impurities	Generic Digital Camera	Color	________	The MII content estimated with was determination coefficient (R2) of 0.996	( Gila *et al.*, 2020)
Coffee trees	Estimate the total amount of cherry coffee beans with direct measurements in the field	Camera Phone	Color	CNN	The CV system achieved 0.594 precision and 0.669 cherry beans correctly classified	( Rodríguez *et al.*, 2020)
Black tea	Evaluation of fermentation degree by FT-NIR and computer vision	Digital Camera	Color and UV–Vis spectrometer	LDA, PCA, and SVM	The mid-level fusion SVM model based on PCA obtained an accuracy of 100%	( Jin *et al.*, 2020)
Table grapes (Italia and Victoria)	Non-destructive and contactless evaluation between fully marketable and residual quality levels	CCD	Color	Random forest models	Accuracy between 92% and 100% was obtained using the binary classification Mmodel by Random Forest	( Cavallo *et al.*, 2019)
Coffee beans	Recognition of coffee roasting degree using color patterns in CIE L*a*b* and grayscale comparing them with the numerical scale of roasting defined	Digital Camera	Color	ANN	The ANN obtained a degree of approval of the toast index with a R2 factor of 0.99	( Leme *et al.*, 2019)
Egg	Estimation of volume and mass of egg with the method disc without damaging the egg.	Portable webcam	size and area	ANNOVA	The CVS with the method used got a result significant of 0.955 y 0.982 for the volume and mass, respectively	( Widiasri *et al.*, 2019)
Fruits/vegetables (Orange, Lemon, Sweet Lime, and Tomato)	A binary classification (Bad/Good) of fruits and vegetables using soft computing techniques	Digital Camera	Color and texture	PCA, BPNN, and PNN	A classification pressure was obtained for the test set of 90.58%, 92.90%, 92.90%, and 89.23% for Lemon, Orange, Sweet Lime, and Tomato, respectively	( Veeranagouda Ganganagowdar & Gundad, 2019)
Patata	Quality classification based on deformity assessment and mass prediction	CCD	Size, form, volume, and surface gradient distribution	PCL, Model 3D	The success rate in mass classification reached 90%. They demonstrated the mass-volume relationship, mass prediction accuracy reached of 7.7 g for MAE and 4.4% for MPE	( Su *et al.*, 2018)
Broiler weight	Broiler weight estimation with the use of a CVS and ANN	Digital Camera	Area, perimeter, convex area, major, minor, and eccentricity	ANN - Bayesian regulation	The model used get a R2 value of 0.98 in the prediction of broiler weight with an accuracy of less than 50 g	( Amraei *et al.*, 2017)
Thomson oranges	Automated and non-intrusive estimation of the pH value use of hybrid ICA-ANN algorithm	Digital Camera	Length, width, area, eccentricity, perimeter, RGB value, contrast, texture, and roughness	ANN, ICA, PCA, MSE, RSM	The hybrid algorithm accuracy determined the pH value obtaining an R2=0.843±0.043	( Sabzi & Arribas, 2018)
Pork loin	Prediction of quality using an online computer vision system with an integrated artificial intelligence model	Industrial Digital Camera	Color	ANN	The results obtained with the CVS was a prediction accuracy of 92.5% for pork color and 75.0% for pork marbling score	( Sun *et al.*, 2018)

## 5. Texture analyzer

The texture of a food is perceived through the response to the contact between the body part and the food. It is a determining characteristic in the acceptance of the product by the consumer ( Civille, 2011; Liu *et al.*, 2019; Muthukumarappan & Karunanithy, 2021). Texture is a quality attribute used in the food industry ( Torres Gonzalez *et al.*, 2015), allowing the parameterization and standardization of food products ( Liu *et al.*, 2019). For example, freshness, a determining characteristic in selecting a vegetable or fruit, can be described by its hardness ( Liu & Zhang, 2021). The latter is one of the primary properties of texture, as well as cohesiveness, viscosity, elasticity, and adhesiveness ( Foegeding *et al.*, 2011).

To determine some of the main textural characteristics mentioned, Friedman in 1963 established a method called *Texture Profile Testing* (TPA) ( Nishinari *et al.*, 2019). This method generates characteristic curves from the force measurement performed by the jaw to realize a change in the geometrical property of the product, generating deformation or fracture ( Kohyama, 2020; Peleg, 2019). The study of these curves allows for establishing and quantifying texture characteristics such as: brittleness, hardness, adhesiveness, cohesiveness, elasticity, gumminess, and chewiness ( Nishinari *et al.*, 2019).

For the measurement of texture characteristics, different methodologies and instruments have been developed, the most widely used technology is centered on texture analyzers or texturometers ( Torres Gonzalez *et al.*, 2015), which are based on the TPA principle, this device simulates the bite of the jaw in two cycles (compression and decompression), through a controlled mechanism that vertically displaces a uniaxial compression cell ( Peleg, 2019). When the cell comes into contact with the product, it generates an electrical signal conditioned by a transducer and sent to a computer to be read by operating software ( Taniwaki & Kohyama, 2012). The displacement is carried out until it reaches either a distance threshold or a force level defined by the operator. When this limit is exceeded, the cell moves back and repeats the cycle ( Liu *et al.*, 2019), simulating the chewing process. Chewing is the first step in the digestion process, and this seeks to prepare food for swallowing. During this process, saliva moistens the chewed food, generating a bolus, which is reduced in size so it can be swallowed. Additionally, saliva helps release flavors and perceive the texture of the food. To achieve this, the intervention of teeth, tongue, saliva, cheeks, and palate is required ( Pereira *et al.*, 2007).

### 5.1 Texture Analyzer Internal Structure

The texture analyzer usually has three fundamental parts: a moving beam, a load cell, and a control panel ( Schmidt, 2018). The first part has a mechanical system that performs the precise vertical displacement of the beam where the load cell is supported; these mechanisms work with a spindle-type system, which has a motor coupled to it that transmits the controlled circular motion ( Sussex, 2013). The load cells are electrical elements that generate a voltage signal when they come into contact with a surface ( Liu *et al.*, 2019). The cells used are in a range of operation from 100 g to 500 kg ( Schmidt, 2018; Sussex, 2013), which will depend on the design of each manufacturer's analyzer.

With the basic structure of the texture analyzer already mentioned, a variety of probes can be incorporated, which, coupled with the load cell, make it possible to measure a large part of the common texture parameters in foodstuffs ( Liu *et al.*, 2019). Among which are the cylindrical probe, which was used to determine the firming kinetics of breadcrumbs ( Jekle *et al.*, 2018). The conical probe that allowed me to measure the texture for deep-fried and air-fried French fries ( Gouyo *et al.*, 2020), The Spherical probe with which they analyzed the texture of the surface of cured ham ( Fulladosa *et al.*, 2021). Also, there are gel and cut probe, each with properties to perform certain texture tests.

### 5.2 Texture analyzer applications

Some applications in which the texture analyzer is used are evidenced in investigations such as the one conducted by Aguirre *et al.* (2018), where texture attributes were validated in the “woody breast” and “cooking methods on the marination” (marinated breast), for which a texture analyzer (TA. XT plus, Texture Technologies, Hamilton, MA) was used. The results were compared with a descriptive test, finding a significant difference in 9 of the 11 texture attributes. Another application is shown in the research conducted by Jiménez *et al.* (2017), where two lionfish surimi patties were studied to validate the efficiency of high-power ultrasound on textural properties. The measurement was performed with a texture analyzer (TA. XT plus, Texture Technologies, Hamilton, MA) correlated with trained panelists.

Other relevant studies, such as those mentioned above, where the aim is to characterize products and correlate them with sensory tests using a texture analyzer, are shown in Table 5.

**Table 5.** Results of relevant studies using TPA in in the food industry.

Food	Purpose of the analysis	Texture analyzer	Type of analysis	Reference
Oleogels	To produce oleogels based on non-germinated and germinated wheat starches with orange essential oil, to replace hydrogenated vegetable fat in bread, and assess the antifungal action.	TA-XT plus (Stable Micro System, UK)	ANOVA and Tukey’s test	( Tavares da Silva *et al.*, 2023)
Pea	To provide a method to improve the effect of microbial transglutaminase-cross-linked pea protein.	TA-XT plus (Stable Micro System, UK)	ANOVA	( Liu *et al.*, 2023)
*Santalum album* essential oil	To evaluate chitosan with sandalwood ( *Santalum album*) essential oil (SEO) as an active packaging film using malic acid as a solvent.	TA-XT plus (Stable Micro System, UK)	ANOVA) and the Tukey Post Hoc test	( Flórez *et al.*, 2022)
Ricotta cheese	To produce edible film made from grey triggerfish gelatin enriched with *M. oleifera* extract as an alternative to synthetic plastic packaging materials, in the dairy products industry.	Texturometer (Lloyd Instruments Ltd., West Sussex, UK)	ANOVA and Duncan’s multiple range test	( Mezhoudi *et al.*, 2022)
Quinoa	Characteristics of Quinoa Starch (TPA)	TA. XT 2i	ANOVA and LSD	( Wu *et al.*, 2017)
Bread	Evaluation of texture attributes	TA. XT plus	ANOVA, LSD, and PCA	( Aleixandre *et al.*, 2021)
Olives	Identification of kinesthetic properties of olives	TA. XT plus	ANOVA	( Lanza & Amoruso, 2018)
Pear	Identification of textural properties of Asian pear peel	TA. XT 2i	ANOVA	( Pham & Liou, 2017)
Strawberry jam	Relationship between sensory and instrumental analysis for the texture of strawberry jam	TA. XT 2i	ANOVA	( Kurotobi *et al.*, 2018)
French fries	Evaluation of the texture of French fries from various restaurants.	TA. XT plus	ANOVA	( Li *et al.*, 2020)
Cooked rice	Identification of textural properties	TA. XT plus	ANOVA, PCA	( Tao *et al.*, 2020)
Chicken breast	Identification of textural properties	TA. XT plus	ANOVA	( Aguirre *et al.*, 2018)

## 6. Electromyographic analysis

Although TPA is a method that simulates the chewing process, its shear rate is low compared to that of the human bite ( Nishinari & Fang, 2018). Therefore, some researchers have focused on finding other mechanisms that allow an understanding of the bite processes of people in a real environment. One of the alternatives is the study of Electromyographic (EMG) signals, which are produced by the nervous system so that the muscles involved during the chewing process react in a certain way producing electrical signals that can be measured ( Besomi *et al.*, 2020; Pereira de Caxias *et al.*, 2021). These signals are captured with an electromyograph, which integrates an instrumentation amplifier that captures and amplifies the EMG signal with the help of three reference electrodes ( Fang *et al.*, 2020), measuring the activity of the jaw muscles and the coordination between them, as well as the movement of the jaw. This signal is sent through a data acquisition board (DAQ), to a processing system where it is processed and sent to a data acquisition system (DAS) ( Gohel & Mehendale, 2020) to a processing system where it is subjected to extraction methods that perform the analysis of the signal ( Ahsan *et al.*, 2009; Zabala *et al.*, 2019). Sodhi *et al.* (2019) correlated bite EMG signals with texture variables (instrumental and sensory) of seven Indian sweets, identifying EMG parameters that distinguish the different textured foods. In addition, the PCA determined the significant correlation between hardness (instrumental and sensory) and sensory stickiness. Similarly, Shimada *et al.* (2012) established intraoral force recordings to analyze the mechanics of human chewing by measuring the force (using strain gauges located on the molars) and the EMG signals (using electrodes located on the masseter muscle) during the biting process of five different products (rice, bread, almonds, banana, and apple). Other relevant studies where the effectiveness of the analysis of EMG signals to determine the texture of a food matrix is sought to be validated are shown in Table 6.

**Table 6.** Results of relevant studies on the relationship between EMG and food texture.

Food	Purpose of the analysis	Instrument	Type of analysis	Results	Reference
Dark chocolate (36%, 70%, and 85% cocoa)	To captured facial EMG over the corrugator and zygomaticus muscles during the consumption of dark chocolate samples (36%, 70%, and 85% cocoa), for to find relation with bitterness perception, linked to cocoa, or hedonic evaluation.	Own EMG	Friedman test	The results suggest that for dark chocolate samples corrugator activity can be linked with hedonic liking.	( Wagner *et al.*, 2023)
7 different foods (Rasgulla, gulab jamun, cham, milk cake, petha, chana murgi, chocolate barfi)	Correlation of EMG variables with texture parameters	Own EMG	PCA	The PCA variables explain 76% of the variance, and the principal components are correlated with instrumental and sensory hardness.	( Sodhi *et al.*, 2019)
Hydrocolloid gels	Identification of different textures	EMG	ANOVA	Identification of the relationship of EMG signals with chewing stress, fracture toughness, and adhesiveness.	( Kohyama *et al.*, 2015)
Dhokla, paneer, rasgulla, cake and jelly	To study the relationship of EMG variables with sensory and instrumental texture parameters.	EMG and texture analyzer	PCA	Fifteen EMG variables were found to be effective in explaining significant texture variation (p ≤ 0.05).	( Rustagi *et al.*, 2022)
Steamed rice cake	Study of rice cake structure with different rice flour particle sizes.	EMG and texture analyzer	TSD, ANOVA and MFA	The EMG response measured the relationship between the chewing process and textural properties.	( Lee *et al.*, 2021)
Brown rice and wheat flour crackers	Physicochemical and textural evaluation	EMG	PCA	Correlation between sensory parameters and EMG, for the two cookies found significant differences (p < 0.05) that distinguish the texture of the cookies.	( Dhillon *et al.*, 2021)

## 7. Acoustic analysis

Food products have the characteristic that when consumed they generate sounds that allow identifying or relating some textural properties such as hardness, crispness, and crunchiness to it ( Dias-Faceto *et al.*, 2020). These sensory properties are related to the freshness of the food. When food is ingested, sound waves are generated that can be perceived through the ears by air conduction or through the jaw by bone conduction. Crispy foods, having a more fragile structure, generate high-frequency sounds; while crunchy foods produce low frequency sounds ( Tunick *et al.*, 2013). Some of the equipment to perform these measurements use devices such as microphones connected to computers o texture analyzers integrated with microphones ( Dias-Faceto & Conti-Silva, 2022), and alternative designs with oscillating tips and piezoelectric sensors ( Taniwaki *et al.*, 2006). All these devices allow capturing the acoustic waves produced by the deformation of the product. These sound waves are captured by microphones which are made up of a diaphragm, a grille, and a transducer. The transducer is responsible for converting the movement generated in the membrane (by detecting a sound wave) into electrical signals. Additionally, it has electronic circuits that help manipulate and improve the electrical signal, among these elements are Light Emitting Diodes (LEDs) whose function is to indicate an on/off state, buttons to manipulate the volume amplitude, filters, among others. This electrical signal is sent to a computer where software analyzes and graphs it ( Dias-Faceto *et al.*, 2020). In humans, the sound wave reaches the outer ear, where the sound is collected and transmitted to the middle ear through the ear canal. Between the outer ear and the middle ear is the tympanic membrane or eardrum, which vibrates when it detects sound (behavior imitated by the microphone diaphragm). The middle ear, made up of the tympanic cavity, houses the auditory ossicles (malleus, incus, and stapes), which transform high-amplitude, low-intensity sound waves into low-amplitude, high-intensity vibrations (behavior similar to that of amplifiers). In this way, the ossicles are the intermediaries in the transmission of vibrations from the eardrum to the inner ear. The inner ear detects and transmits auditory impulses to the brain (function carried out by the software housed in the computer) through the vestibulocochlear nerve (function carried out by a transmission medium such as cables) ( Duizer, 2001). It is known that soft tissues have a damping effect (a function performed by attenuating circuits) on the sound produced when chewing food. To match what is heard during the consumption of a product, bone-borne noise must be attenuated at a frequency of 160 Hz, while air-borne sound must be attenuated at 160 Hz and amplified at 3.5 kHz ( Dacremont Colas & Sauvageot, 1991). Due to these differences in sound contribution, the two sounds must be combined and equalized to fully quantify the acoustic sensations perceived during the consumption of crunchy or crispy products ( Vickers & Bourne, 1976).

Researchers such as Błońska *et al.* (2014), showed that adding inulin with reduced fat content significantly affected the acoustic parameters of Short-Dough Biscuits. Eight Short-Dough Biscuits with different percentages of inulin addition were compared, determining the impact on the acoustic properties and the decrease in the breaking workforce. For example, the biscuit with 74.1% fat and 18.5% inulin, showed a low acoustic energy level of 1.134 a. u. this compared to a biscuit with 55.6% fat and 9.3% inulin, in which a high acoustic energy level of 17.373 a. u. was found, the former being less brittle and hard compared to the latter. This was achieved using a Zwick 1445 measuring system (Zwick GmbH & Co. KG, Ulm, Germany). Separately, Jakubczyk *et al.* (2017) studied the acoustic signals generated during puncture tests on some coextruded cereal products with different fillings (toffee, milk, fruit jelly, coconut, and chocolate creams), to perform the analysis of hardness, crunchiness, and texture sound attributes for each product. The results showed that the snacks with jelly filling were perceived as less crunchy and soft, compared to the snack with milk cream filling, which showed high acoustic and mechanical values that link it to crunchiness. The variables were measured with a BC45 cooking extruder (Clextral, Firminy, France). Other relevant investigation, such as those mentioned above, where acoustic analysis was performed to determine some textural properties of certain foods, can be seen in Table 7.

**Table 7.** Results of relevant studies on the relationship between acoustic analysis and texture of food.

Food	Purpose of the analysis	Instrument	Type of analysis	Results	Reference
Chips, cereals, cookies, others.	Identification of instrumental configuration with increased sensitivity of acoustic signals used as a sensory indicator of dry and crispy foods.	TA. XT plus Texture Analyzer	SPL Dias-Faceto, Salvador, and Conti-Silva 2020	Identification of gain 1 as the most suitable acoustic condition to define different croaking intensity.	( Dias-Faceto *et al.*, 2020)
Apple, cookie, biscuit, and potato chip	Acoustic measurement of food texture	Designed instruments, Swing arm	FFT and ETI	Identification of textures for each product with a confidence level of 95%	( Akimoto *et al.*, 2019)
Apple, biscuit, cucumber, lettuce, Japanese cracker, and radish	Acoustic vibration measurement for food texture determination	Device with piezoelectric sensor in a horizontal manner	FFT and ETI	Determination of different texture indices according to device response.	( Iwatani *et al.*, 2013)
Banana, salad, rice balls, others	Estimation of food texture	Vibraudio EM20 Microphone	SOM	A model was obtained to predict texture with 90% accuracy.	( Zhang *et al.*, 2012b)

## 8. Other considerations

Other considerations to take into account when acquiring a technological tool for the study of food matrices are:

• Costs: The acquisition of technological tools, such as those presented in this article, can be expensive. For example, the cost of e-nose ranges from USD 100 to USD 1000; the texturometer at an approximate value of USD 23900; a colorimeter at an approximate value of USD 12000 and an electromyograph (for medical use) at an approximate value of USD 10560. Regarding accessories and/or additional components, these can vary between USD 10 and USD 2300. This aspect must be considered when purchasing any of these technological tools to keep them operational.
• Maintenance and calibration: After a period of use, these technological tools, like any equipment, will require periodic maintenance and regular calibration to ensure their accuracy and reliability. These costs can range from 3% to 20% of the initial value of the equipment per year.

Finally, it must be considered that the study of various food matrices is characterized by the variability of the samples, this being biological material. Thus, some e-tongues and e-noses are designed to identify specific compounds with high precision, thus limiting the analysis of food matrices for which they were not designed. Regarding the use of the colorimeter, lighting conditions and texture of the food matrix can affect the results. All of the above shows the economic and technical limitations that these technological tools may have.

## 9. General conclusions

As evidenced in this review, some technological tools have been developed to emulate the functioning of the five senses (smell, taste, sight, touch, and hearing), seeking to quantify and characterize some sensory properties of different food matrices, to compare, parameterize and standardize a product. These investigations show that the use of technological tools guarantees the repeatability and reproducibility of the process, compared to the results obtained when working with trained panelists. Therefore, the use of this type of device reduces the number of samples required to perform the analysis, in addition to dispensing with the need for a team of trained panelists, which generates a reduction in costs. In addition, another advantage of these tools is the wider measurement capacity compared to that of human beings. However, most of the tools analyzed only have the property of measuring a single characteristic in a food matrix, this becomes an inconvenience when it comes to characterizing an entire product, for which many tools must be available, samples required and therefore an increase in the time of the analysis and availability of personnel to carry out the process. This is why both the scientific community and the industry, increasing the development of research that seeks to create new technological tools that allow the measurement of two or more sensory characteristics in a food matrix. All the above, seeking to develop new food products and improve existing ones to satisfy the sensory experiences of the consumer, driving growth in the food sector.

## Data availability

No data are associated with this article.
