# Supplementary material for: Technological tools for the measurement of sensory characteristics in food: A review
Source: F1000Res. 2024 Feb 1;12:340. Originally published 2023 Mar 27. [Version 2] doi: 10.12688/f1000research.131914.2 (PMC10844804; doi:10.12688/f1000research.131914.2)
Supplement: Peer review history

# Reviewer report: Reviewer response for version 2

**DOI:** 10.5256/f1000research.159245.r243292

**Published:** 2024-02-09

**Authors:** Mustapha Muhammad Nasiru

**Affiliations:** 1 Department of Food Science and Technology, Federal University Dutsin-Ma, Dutsin-Ma, Katsina, Nigeria

Approved as there are no further comments.

Is the review written in accessible language?

Yes

Are all factual statements correct and adequately supported by citations?

Yes

Are the conclusions drawn appropriate in the context of the current research literature?

Yes

Is the topic of the review discussed comprehensively in the context of the current literature?

Yes

Reviewer Expertise:

Food Processing and Preservation

I confirm that I have read this submission and believe that I have an appropriate level of expertise to confirm that it is of an acceptable scientific standard.

# Reviewer report: Reviewer response for version 2

**DOI:** 10.5256/f1000research.159245.r243290

**Published:** 2024-02-05

**Authors:** Zahir Al-Attabi

**Affiliations:** 1 Department of Food Science and Nutrition, College of Agricultural and Marine Sciences, Sultan Qaboos University, Al-Khodh, Muscat Governorate, Oman

Authors responses are acceptable.

Is the review written in accessible language?

Yes

Are all factual statements correct and adequately supported by citations?

Yes

Are the conclusions drawn appropriate in the context of the current research literature?

Partly

Is the topic of the review discussed comprehensively in the context of the current literature?

Partly

Reviewer Expertise:

Food sensory, food analysis, gas chromatography, E-nose

I confirm that I have read this submission and believe that I have an appropriate level of expertise to confirm that it is of an acceptable scientific standard.

# Reviewer report: Reviewer response for version 1

**DOI:** 10.5256/f1000research.144802.r200391

**Published:** 2023-09-19

**Authors:** Mustapha Muhammad Nasiru

**Affiliations:** 1 Department of Food Science and Technology, Federal University Dutsin-Ma, Dutsin-Ma, Katsina, Nigeria

This review explores various technological tools, including e-nose, e-tongue, artificial vision systems, and texture analysis instruments used to analyse and assess food properties like quality, composition, maturity, authenticity, and origin. By standardising these characteristics, they enhance existing food products and create new ones that cater to consumers' sensory preferences. This advancement supports growth in the food sector by delivering satisfying sensory experiences to consumers.

 The paper exhibits a well-organised structure, with coherent headings and subheadings that contribute to the overall clarity of the manuscript. The write-up is satisfactory and presented in good English. The figures and tables provided are clear and comprehensible, aiding in understanding the findings.

 However, there are some suggestions as follows:

- The abstract should be rewritten to include the findings of the study.
- Colour measurement devices were not discussed sufficiently, so more discussion is needed.

Is the review written in accessible language?

Yes

Are all factual statements correct and adequately supported by citations?

Yes

Are the conclusions drawn appropriate in the context of the current research literature?

Yes

Is the topic of the review discussed comprehensively in the context of the current literature?

Yes

Reviewer Expertise:

Food Processing and Preservation

I confirm that I have read this submission and believe that I have an appropriate level of expertise to confirm that it is of an acceptable scientific standard, however I have significant reservations, as outlined above.

# Response

**Published:** 2023-10-26

**Authors:** Claudia Garzón

**Affiliations:** Universidad de La Sabana, Colombia

We thank the reviewer for the valuable comments made on version 1 of the manuscript. We answer the reviewers’ comments as follows: 

  

1. The abstract should be rewritten to include the findings of the study.
  Answer. The abstract was adjusted. 
2. Colour measurement devices were not discussed sufficiently, so more discussion is needed. 
  Answer. We have added sub-section 4.1which talks about the colorimeter. 

 

# Response

**Published:** 2023-11-21

**Authors:** Jose martinez

**Affiliations:** Universidad de La Sabana, Colombia

We thank the reviewer for the valuable comments made on version 1 of the manuscript. We answer the reviewers’ comments as follows:

 1. The abstract should be rewritten to include the findings of the study. 

**Answer. **The abstract was adjusted. 

  2. Colour measurement devices were not discussed sufficiently, so more discussion is needed. 

**Answer.** We have added sub-section 4.1which talks about the colorimeter.

# Reviewer report: Reviewer response for version 1

**DOI:** 10.5256/f1000research.144802.r200385

**Published:** 2023-09-11

**Authors:** Zahir Al-Attabi

**Affiliations:** 1 Department of Food Science and Nutrition, College of Agricultural and Marine Sciences, Sultan Qaboos University, Al-Khodh, Muscat Governorate, Oman

The manuscript described several technologies applied in the field of food sensory. The flow of the manuscript is good and well-structured. The information provided is useful. However, some of these technologies are extensively reviewed like e-nose and e-tongue.

 The following would improve the manuscript:

1. GC and GC/O were mentioned in the introduction but were not discussed.
2. Highlighting the advances in these technologies. 
3. The colorimeter was not discussed. 
4. Discuss the correlation between human sensory evaluation and these technologies.
5. Disadvantages of these technologies.

Other minor comments are amended in the manuscript PDF file linked.

Is the review written in accessible language?

Yes

Are all factual statements correct and adequately supported by citations?

Yes

Are the conclusions drawn appropriate in the context of the current research literature?

Partly

Is the topic of the review discussed comprehensively in the context of the current literature?

Partly

Reviewer Expertise:

Food sensory, food analysis, gas chromatography, E-nose

I confirm that I have read this submission and believe that I have an appropriate level of expertise to confirm that it is of an acceptable scientific standard, however I have significant reservations, as outlined above.

# Response

**Published:** 2023-10-26

**Authors:** Claudia Garzón

**Affiliations:** Universidad de La Sabana, Colombia

We thank the reviewer for the valuable comments made on version 1 of the manuscript. We answer the reviewers’ comments as follows: 

1. GC and GC/O were mentioned in the introduction but were not discussed. 

Answer. In session 2 the following technologies were discussed: Gas Chromatography-Olfactometry (GC-O), Gas Chromatography-Mass Spectrometry (GC-MS) and Headspace Solid Phase Microextraction (HS-SPME). 

  2. Highlighting the advances in these technologies.   

 Answer. In session 2 the following technologies were discussed: Gas Chromatography-Olfactometry (GC-O), Gas Chromatography-Mass Spectrometry (GC-MS) and Headspace Solid Phase Microextraction (HS-SPME). 

 3. The colorimeter was not discussed.

 Answer. We have added sub-section 4.1 which talks about the colorimeter. 

 4. Discuss the correlation between human sensory evaluation and these technologies. 

 Answer. We have added in sections 2.1, 3.1, 4.2.1, 5, and 7 information indicating the correlation between human sensory evaluation and the technological tools mentioned in the article. 

  5. Disadvantages of these technologies. 

 Answer. To give scope to this recommendation, we have added section 8.

# Response

**Published:** 2023-11-21

**Authors:** Jose martinez

**Affiliations:** Universidad de La Sabana, Colombia

We thank the reviewer for the valuable comments made on version 1 of the manuscript. We answer the reviewers’ comments as follows: 

 1. GC and GC/O were mentioned in the introduction but were not discussed. 

**Answer. **In session 2 the following technologies were discussed: Gas Chromatography-Olfactometry (GC-O), Gas Chromatography-Mass Spectrometry (GC-MS) and Headspace Solid Phase Microextraction (HS-SPME). 

 2. Highlighting the advances in these technologies.  

**Answer. **In session 2 the following technologies were discussed: Gas Chromatography-Olfactometry (GC-O), Gas Chromatography-Mass Spectrometry (GC-MS) and Headspace Solid Phase Microextraction (HS-SPME). 

 3. The colorimeter was not discussed.  

**Answer.** We have added sub-section 4.1 which talks about the colorimeter. 

 4. Discuss the correlation between human sensory evaluation and these technologies. 

**Answer. **We have added in sections 2.1, 3.1, 4.2.1, 5, and 7 information indicating the correlation between human sensory evaluation and the technological tools mentioned in the article. 

 5. Disadvantages of these technologies. 

**Answer. **To give scope to this recommendation, we have added section 8.

# Reviewer report: Reviewer response for version 1

**DOI:** 10.5256/f1000research.144802.r194983

**Published:** 2023-09-07

**Authors:** Ítala M. G. Marx

**Affiliations:** 1 University of Minho, Braga, Braga, Portugal

The manuscript "Technological Tools for Sensory Analysis in the Food Industry" provides an overview of various technological tools used in the food industry to measure and analyze sensory characteristics of food products. The paper covers electronic nose, electronic tongue, artificial vision systems, texture analyzers, electromyographic analysis, and acoustic analysis.

 This present manuscript provides a valuable overview of technological tools used for sensory analysis in the food industry. It offers practical insights into how these tools can be applied to assess various sensory attributes of food products. However, it would benefit from a more critical evaluation of the limitations and challenges associated with these tools and a discussion of recent advancements in the field. Additionally, a deeper exploration of data analysis methods would enhance the paper's utility for researchers and practitioners in the food industry.

 This article does not demonstrate an innovative character. There are several reviews already published in this area. This manuscript should draw attention to real innovations and aspects that have not yet been addressed in the literature.

 Below I detail some positive aspects of the review, but also some points that must be improved so that this manuscript can be accepted.

 The paper provides a comprehensive overview of various technological tools used in sensory analysis in the food industry. It covers a wide range of devices and techniques, including e-nose, e-tongue, artificial vision systems, texture analyzers, electromyographic analysis, and acoustic analysis. This comprehensive coverage is valuable for readers interested in understanding the diversity of tools available for sensory analysis.

 This manuscript is well-structured, with each section dedicated to a specific technological tool. This organization makes it easy for readers to navigate and find information on each tool separately. Additionally, the internal structure and applications of each tool are described in detail, providing a clear understanding of their operation and potential uses.

 The authors emphasize the practical applications of these technological tools in the food industry. It highlights how these tools can be used to assess various sensory characteristics such as flavor, texture, and appearance. The practical examples and applications mentioned in the paper demonstrate the real-world utility of these tools for quality control and product development.

 However, the authors should provide a wealth of information on the various tools, it lacks critical evaluation and discussion of their limitations and challenges. A **review** should not only highlight the strengths but also address potential weaknesses and constraints associated with the use of these tools. For instance, the paper does not discuss the cost, maintenance requirements, and calibration challenges that may arise when implementing these tools in food production settings. The manuscript does not discuss recent advancements and developments in the field of sensory analysis technology. Given the rapid pace of technological innovation, it would be beneficial to include information on any emerging tools or techniques that have been developed since the paper's publication.

 Finally, the authors briefly mention some data analysis methods used in conjunction with these tools, such as Principal Component Analysis (PCA) and machine learning classifiers. However, it would be helpful to provide more in-depth discussions on data analysis techniques and how they are applied to interpret the results obtained from these tools.

Is the review written in accessible language?

Yes

Are all factual statements correct and adequately supported by citations?

Partly

Are the conclusions drawn appropriate in the context of the current research literature?

Partly

Is the topic of the review discussed comprehensively in the context of the current literature?

Partly

Reviewer Expertise:

Olive oil; Bioactive compounds; Phenolic compounds; Emerging technologies of phenols extraction; electrochemical sensor devices; e-tongue; e-nose; chromatography.

I confirm that I have read this submission and believe that I have an appropriate level of expertise to confirm that it is of an acceptable scientific standard, however I have significant reservations, as outlined above.

## References

[1] : Recent technology for food and beverage quality assessment: a review. *J Food Sci Technol* .2023;60(6) : 10.1007/s13197-022-05439-8 1681-1694 10.1007/s13197-022-05439-8 35463865 PMC9014778

[2] : Applications of electronic nose (e-nose) and electronic tongue (e-tongue) in food quality-related properties determination: A review. *Artificial Intelligence in Agriculture* .2020;4: 10.1016/j.aiia.2020.06.003 104-115 10.1016/j.aiia.2020.06.003

[3] : Electronic noses and tongues: applications for the food and pharmaceutical industries. *Sensors (Basel)* .2011;11(5) : 10.3390/s110504744 4744-66 10.3390/s110504744 22163873 PMC3231405

[4] : Toward the Development of Combined Artificial Sensing Systems for Food Quality Evaluation: A Review on the Application of Data Fusion of Electronic Noses, Electronic Tongues and Electronic Eyes. *Sensors (Basel)* .2022;22(2) : 10.3390/s22020577 10.3390/s22020577 35062537 PMC8778015

[5] : Potential use of electronic noses, electronic tongues and biosensors as multisensor systems for spoilage examination in foods. *Trends in Food Science & Technology* .2018;80: 10.1016/j.tifs.2018.07.018 71-92 10.1016/j.tifs.2018.07.018

# Response

**Published:** 2023-11-21

**Authors:** Claudia Garzón

**Affiliations:** Universidad de La Sabana, Colombia

We thank the reviewer for the valuable comments made on version 1 of the manuscript. We answer the reviewers’ comments as follows: 

 1. However, the authors should provide a wealth of information on the various tools, it lacks critical evaluation and discussion of their limitations and challenges. A review should not only highlight the strengths but also address potential weaknesses and constraints associated with the use of these tools. For instance, the paper does not discuss the cost, maintenance requirements, and calibration challenges that may arise when implementing these tools in food production settings.   

**Answer.** To give scope to this recommendation, we have added section 8. 

  2. The manuscript does not discuss recent advancements and developments in the field of sensory analysis technology. Given the rapid pace of technological innovation, it would be beneficial to include information on any emerging tools or techniques that have been developed since the paper's publication.  

**Answer.** Thanks for the recommendation. We have added a new table that contains works where the colorimeter is used to characterize food matrices (table 3). Additionally, we add works developed between 2022 to date in tables 1, 2, 4, 5 and 6.   

  3. Finally, the authors briefly mention some data analysis methods used in conjunction with these tools, such as Principal Component Analysis (PCA) and machine learning classifiers. However, it would be helpful to provide more in-depth discussions on data analysis techniques and how they are applied to interpret the results obtained from these tools.  

**Answer.** We appreciate the suggestion made; however, this review is focused on presenting some of the technological tools used for the analysis of sensory characteristics in food matrices. Therefore, data analysis methods used in conjunction with these tools, such as Principal Component Analysis (PCA) and machine learning classifiers, were not discussed in this review. However, we are working with a master's student on a review article which includes the compilation of research studies related to existing data analysis methods that have been used in the study of food matrices.

# Response

**Published:** 2023-11-21

**Authors:** Jose martinez

**Affiliations:** Universidad de La Sabana, Colombia

We thank the reviewer for the valuable comments made on version 1 of the manuscript. We answer the reviewers’ comments as follows: 

 1. However, the authors should provide a wealth of information on the various tools, it lacks critical evaluation and discussion of their limitations and challenges. A **review** should not only highlight the strengths but also address potential weaknesses and constraints associated with the use of these tools. For instance, the paper does not discuss the cost, maintenance requirements, and calibration challenges that may arise when implementing these tools in food production settings.  

**Answer. **To give scope to this recommendation, we have added section 8. 

 2. The manuscript does not discuss recent advancements and developments in the field of sensory analysis technology. Given the rapid pace of technological innovation, it would be beneficial to include information on any emerging tools or techniques that have been developed since the paper's publication. 

**Answer. **Thanks for the recommendation. We have added a new table that contains works where the colorimeter is used to characterize food matrices (table 3). Additionally, we add works developed between 2022 to date in tables 1, 2, 4, 5 and 6.   

 3. Finally, the authors briefly mention some data analysis methods used in conjunction with these tools, such as Principal Component Analysis (PCA) and machine learning classifiers. However, it would be helpful to provide more in-depth discussions on data analysis techniques and how they are applied to interpret the results obtained from these tools. 

**Answer**. We appreciate the suggestion made; however, this review is focused on presenting some of the technological tools used for the analysis of sensory characteristics in food matrices. Therefore, data analysis methods used in conjunction with these tools, such as Principal Component Analysis (PCA) and machine learning classifiers, were not discussed in this review. However, we are working with a master's student on a review article which includes the compilation of research studies related to existing data analysis methods that have been used in the study of food matrices.
